# Stearic acid/fumed silica/Fe_3_O_4_ composite phase change materials with low thermal conductivities and magnetically accelerated heating performance for wearable thermotherapy†

**DOI:** 10.1039/d5na00133a

**Published:** 2025-04-09

**Authors:** Giang Tien Nguyen, Van Khanh Thi Nguyen, Thanh Sang Nguyen

**Affiliations:** a Faculty of Chemical and Food Technology, Ho Chi Minh City University of Technology and Education (HCMUTE) 1 Vo Van Ngan, Thu Duc Ho Chi Minh City 700000 Vietnam ntgiang@hcmute.edu.vn

## Abstract

Accelerating heating rates while maintaining low thermal conductivities (TCs) of fumed silica (FS)-based composite phase change materials is essential to achieve perfect thermotherapy materials. In this work, FS was combined with stearic acid (SA) and Fe_3_O_4_ nanoparticles (NPs) to form magnetic composite phase change materials (MCPCMs) with low TCs and effective magnetic heating performance. The MCPCMs were investigated with a fixed content of SA (70%) and varying contents of FS (20–30%) and Fe_3_O_4_ NPs (0–10%). The prepared MCPCMs exhibited suitable exothermic temperatures (∼51 °C) and high energy storage capacities (∼132 J g^−1^) accompanied by high crystallization fractions (>93%). The low TCs of FS and Fe_3_O_4_ NPs resulted in a decrease in TCs for the prepared MCPCMs of 31.8–45.4% compared to pure SA. In addition, the prepared MCPCMs inherited superparamagnetism from Fe_3_O_4_ NPs, which led to an effective magnetothermal conversion under an alternating magnetic field. For comparison, MCPCM7 (the prepared MCPCM with 7% Fe_3_O_4_ NPs) exhibited magnetic heating rates 13.0 and 7.5 times faster than heating by using ovens at 100 and 150 °C, respectively. MCPCM7 was used as a heat pad, which could release heat within 42–52 °C for 35 min to a volunteer's body. Last but not least, MCPCM7 possessed high thermal stability and excellent cycling durability for 500 heat storage/release cycles. The above-mentioned properties make it highly promising for practical thermotherapy applications.

## Abstract 2

The prepared SA/FS/Fe_3_O_4_ composite phase change material possesses magnetically accelerated heating performance and high phase change enthalpy, which makes it suitable for thermotherapy applications.

## Graphical abstract

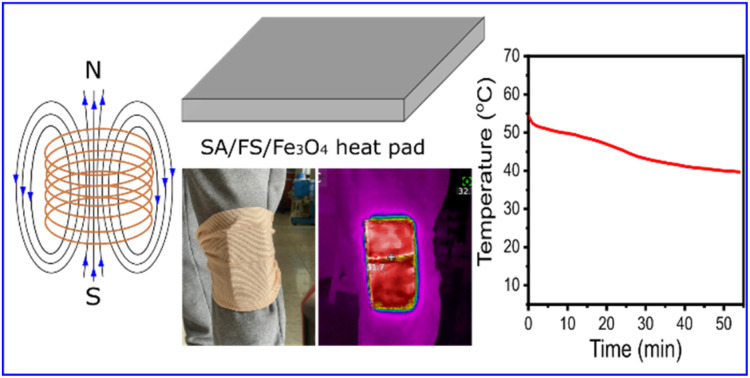

## 1. Introduction

Thermotherapy is a widely used treatment to relieve pain, improve blood circulation, and promote relaxation. It is commonly used for conditions such as arthritis, muscle stiffness, and chronic pain, offering a non-invasive and drug-free approach to healing.^1,2^ Thermotherapy involves applying heat to the body at specific temperatures for controlled durations, categorized as low level (35–40 °C, 6–72 h), middle level (40–50 °C, 15–60 min), and high level (50–55 °C, 4–6 min).^3,4^ However, conventional thermotherapy methods, such as infrared lamps or electric heating pads, struggle to maintain consistent temperatures, posing potential risks.^1,5^

Recently, composite phase change materials (PCMs), particularly PCMs impregnated in porous supports, have gained attention as promising thermotherapy materials due to their ability to store and release a significant amount of heat at a nearly constant temperature during solid–liquid phase transition processes.^6^ They have been fabricated into various forms, including shoulder pads, eyeshades, knee pads, and face masks for the thermotherapy treatment of allergic rhinitis, rheumatoid arthritis, and eye fatigue.^1,7–9^ Meanwhile, the porous supports are crucial in stabilizing PCMs during phase transitions and influencing key properties such as thermal conductivity (TC) and energy conversion. Studies have shown that composite PCMs with low TC can slow heat release during crystallization, prolonging heat retention, thus making them ideal for thermotherapy applications.^9–11^

Fumed silica is a commonly used porous support for PCMs because of its high porosity, high PCM adsorption (up to 80%), inexpensiveness, non-toxicity, and widespread availability.^12–14^ Notably, FS has an extremely low TC of ∼0.05 W (m^−1^ K^−1^) due to its fine pore structure, which restricts convection.^15^ Thus, FS was often used for thermal insulation applications.^16,17^ Previous reports showed that integrating FS with PCMs such as 1-octadecanol,^12^ polyethylene glycol,^18^ and sodium acetate trihydrate^13,14^ reduced TCs by 1.5 to 3.2 times compared to that of pure PCMs. While FS-based composite PCMs hold great promise for thermotherapy due to their low TC, they suffer from a slow heating rate, limiting their heat storage efficiency. Furthermore, conventional heating methods using ovens or hot water present challenges: ovens provide inefficient convective heat transfer, and hot water can degrade FS-based composite PCMs due to high permeability. Researchers have explored electrically heated composite PCMs to improve heating performance, and promising results have been yielded. To enable this function, conductive additives such as expanded graphite,^2,19^ graphene,^20,21^ carbon nanotubes,^22^ Mxenes,^23^ and boron nitride,^24^ have been introduced. However, these additives typically increased TC, possibly decreasing heat release duration. Therefore, developing FS-based composite PCMs with efficient and safe heating capabilities while maintaining low TC is crucial for advancing thermotherapy materials.

Instead of electric heating, in this work, FS-based composite PCMs were equipped with a magnetic heating ability. Specifically, FS was combined with Fe_3_O_4_ NPs and stearic acid (SA) to create magnetic composite phase change materials (MCPCMs). Fe_3_O_4_ NPs, with a superparamagnetic nature, were selected as a heating agent due to the rapid magnetothermal conversion effect of magnetic materials under an alternating magnetic field.^25,26^ In addition, Fe_3_O_4_ NPs have inherently low TC, meaning their inclusion does not significantly increase the TC of composite PCMs.^27,28^ Meanwhile, SA was chosen as the PCM due to its relatively high phase change enthalpy (∼200 J g^−1^), suitable crystalline temperature (∼52 °C), affordability, and non-toxicity. The MCPCMs were formulated with a fixed SA content (70%) and varying FS (20–30%) and Fe_3_O_4_ NP (0–10%) contents to assess leakage resistance, phase change behavior, crystallinity, thermal stability, and TC. Additionally, their magnetothermal conversion and heat storage/release properties were investigated, and their magnetic heating performance was compared to that of traditional oven heating. Finally, a heat pad was fabricated using an MCPCM, and its practical thermotherapy performance was comprehensively evaluated and discussed.

## 2. Experimental section

### 2.1 Materials

Fumed silica (Aerosil 200) was purchased from Evonik Operations (German). Stearic acid (99%), iron(iii) chloride (FeCl_3_, AR), sodium acetate trihydrate (CH_3_COONa·3H_2_O, AR), ethylene glycol (AR), absolute ethanol, and acetone (AR) were purchased from Xilong Chemical (China).

### 2.2 Preparation of Fe_3_O_4_ NPs

The preparation of Fe_3_O_4_ NPs was performed according to the procedure described in our recent publication.^19^ First, 0.54 g FeCl_3_ and 2.3 g CH_3_COONa·3H_2_O were dissolved in 60 mL ethylene glycol using ultrasonication. The solution obtained was then poured into a Teflon-lined autoclave and heated at 200 °C for 10 h. The black powder was obtained by centrifugation and washed with water and acetone to remove impurities and finally dispersed in 30 mL of acetone for later use. To obtain solid Fe_3_O_4_ NPs for characterization, the powder was dried at 120 °C for 24 h instead of dispersing in acetone.

### 2.3 Preparation of magnetic composite phase change materials

The MCPCMs were prepared using a widely used solvent-assisted method.^6^ Appropriate amounts of SA, FS, and Fe_3_O_4_ NPs were added to acetone and stirred at ambient temperature for 2 hours. Then, the mixture was heated to the boiling point of acetone until all acetone completely evaporated. The obtained materials were finally treated at 70 °C for 24 h. Previous reports showed that FS, without added solid additives, could stabilize 70–80% of PCMs without leakage.^13,18,29^ In this work, adding Fe_3_O_4_ NPs led to a decreased FS fraction in the obtained MCPCMs, possibly causing a lower content of stabilized SA. Thus, the MCPCMs were prepared and characterized with a fixed SA content as low as 70%, while Fe_3_O_4_ content varied between 0 and 10%. An increase in Fe_3_O_4_ content resulted in a corresponding decrease in FS content. The prepared MCPCMs were abbreviated as MCPCMX, in which the X stands for Fe_3_O_4_ content in the MCPCM. For example, MCPCM10 means a composite was prepared with 70% SA, 10% Fe_3_O_4_ NPs, and 20% FS. Four MCPCMs were prepared, and the specific constituents are shown in Table 1.

**Table 1:** Specific constituents of prepared MCPCMs

	SA (%)	Fe_3_O_4_ (%)	FS (%)
MCPCM0	70	0	30
MCPCM4	70	4	26
MCPCM7	70	7	23
MCPCM10	70	10	20

### 2.4 Characterization methods

Scanning electron microscopy (SEM) was conducted using a Hitachi S-4800 instrument (Hitachi, Japan). The N_2_ adsorption–desorption isotherm was obtained using a Belsorp-Max instrument (MicrotracBel, Japan). Fourier-transform infrared spectroscopy (FTIR) was conducted using an FTIR 4600 model (JASCO, Japan). X-ray diffraction (XRD) was conducted using an Empyrean Diffractometer (Malvern Panalytical, United Kingdom). Differential scanning calorimetry (DSC) was conducted using a 214 Polyma Instrument (NETZSCH, German) performed under a N_2_ flow of 20 mL min^−1^ and a ramp rate of 5 °C min^−1^. The phase change temperature and enthalpy were calculated as the onset temperature and area under the phase change peak, respectively. Thermogravimetric analysis (TGA) was conducted using a Labsys Evo TG-DSC 1600 analyzer (Setaram Instrument, USA) with a ramp rate of 10 °C min^−1^ and N_2_ flow of 20 mL min^−1^. Thermal conductivity was measured using a TPS 3500 instrument (Hot Disk AB, Sweden). Vibrating sample magnetometry (VSM) was conducted using a Microsens instrument (Microsens, USA). Infrared images were obtained using an infrared camera (Uni-T 260 V, China).

## 3. Results and discussion

### 3.1 Characterization

**Fig. 1:**
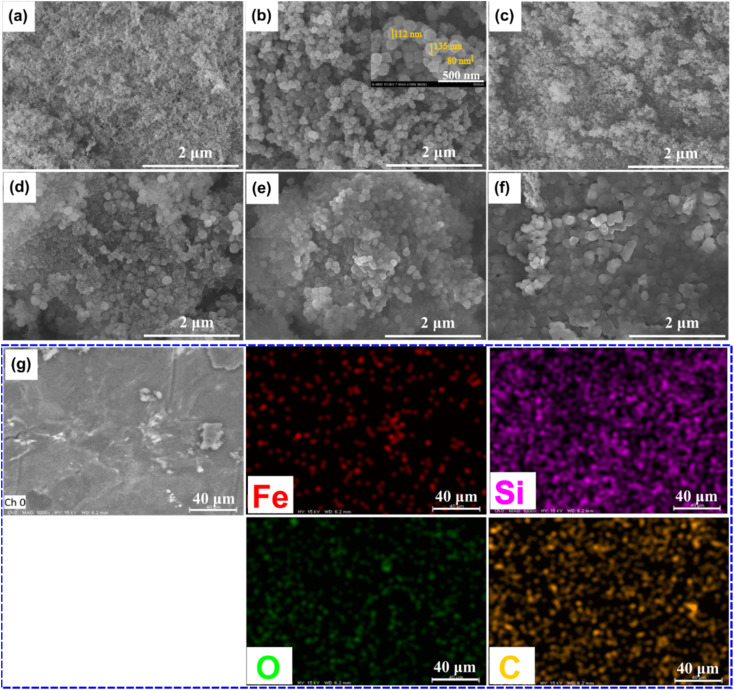
SEM images of FS (a), Fe_3_O_4_ (b), MCPCM0 (c), MCPCM4 (d), MCPCM7 (e), and MCPCM10 (f), and EDS image of MCPCM7 (g).

**Fig. 2:**
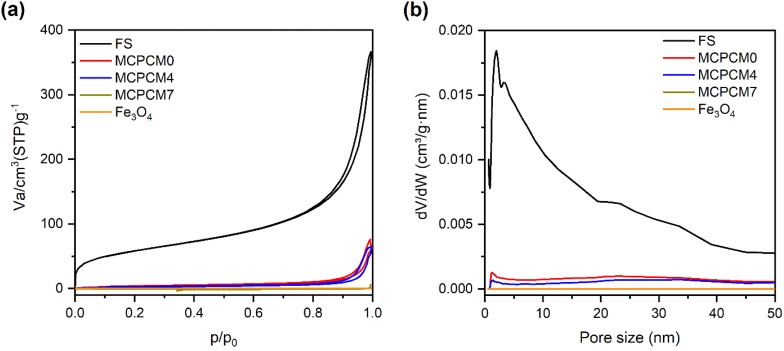
N_2_ adsorption–desorption isotherms (a) and corresponding pore size distribution (b) of FS, Fe_3_O_4_, and the prepared MCPCMs. It is noted that the data of FS were taken from our recent report.^18^

The SEM image of pristine FS is exhibited in Fig. 1a. FS was composed of SiO_2_ nanoparticles aggregated into an interconnected porous structure. The N_2_ adsorption–desorption isotherm of FS (Fig. 2a) presented a surface area of 205 m^2^ g^−1^, a sharp adsorption at a low pressure of *P*/*P*_0_ < 0.1, and a slow adsorption at a pressure of 0.1 < *P*/*P*_0_ < 0.9, suggesting the existence of micropores and mesopores. The pore size distribution (PSD) of FS obtained from the N_2_ sorption isotherm (Fig. 2b) confirmed the presence of micro and mesopores. In addition, sharp adsorption was further observed at the pressure *P*/*P*_0_ > 0.9, attributed to the presence of macropores. Indeed, our previous report demonstrated that FS possessed macropores within 50–150 nm and a total pore volume of 17 m^3^ g^−1^ characterized by mercury intrusion porosimetry.^29^ The interconnected micro-, meso-, and macropores, and large pore volume make FS ideal for the infiltration of PCMs. Meanwhile, the prepared Fe_3_O_4_ consisted of tiny particles aggregated into particles of 80–135 nm (Fig. 1b and S1(a)†) and was a nonporous material characterized by zero N_2_ adsorption and pores (Fig. 2). As shown in the particle size distribution graph (Fig. S1(b)†), the tiny particles were in the range of 13–27 nm and dominant at 17 nm. As reported in previous literature studies,^30,31^ Fe_3_O_4_ NPs exhibited a single-domain state within the size range of 14 to 36 nm. Thus, with a size of 13–27 nm, the prepared Fe_3_O_4_ NPs met the size range for the single-domain state and possessed superparamagnetic properties, as further confirmed by their VSM curve (see Section 3.5).

The SEM images of the prepared MCPCMs are shown in Fig. 1c–f. Except for MCPCM0 (Fig. 1c), the other composites, including MCPCM4, MCPCM7, and MCPCM10 (Fig. 1d–f), showed the presence of Fe_3_O_4_ NPs mixed with FS. Also, the porous structure of FS was filled with SA in all composites. Compared to pristine FS, the prepared MCPCMs exhibited a decline in N_2_ adsorption (Fig. 2a) and corresponding pore intensities (Fig. 2b), further confirming the infiltration of SA into FS pores. With more careful observation, it is seen that the N_2_ adsorption of the MCPCMs increasingly decreased with the Fe_3_O_4_ contents. This could be understood by the replacement of FS with Fe_3_O_4_. In the composites, the N_2_ adsorption was mainly due to the porous structure of FS, while the contribution of Fe_3_O_4_ and SA was insignificant. All composites had the same SA content but varying Fe_3_O_4_ and FS contents. The increase in Fe_3_O_4_ content induced a decrease in FS fraction, as shown in Table 1, causing a more significant reduction in N_2_ adsorption. An EDS image of MCPCM7 (Fig. 1g) exhibited an even distribution of Fe, Si, O, and C elements. All these results demonstrated a successful combination of FS, Fe_3_O_4_, and SA in the form of magnetic composite phase change materials.

**Fig. 3:**
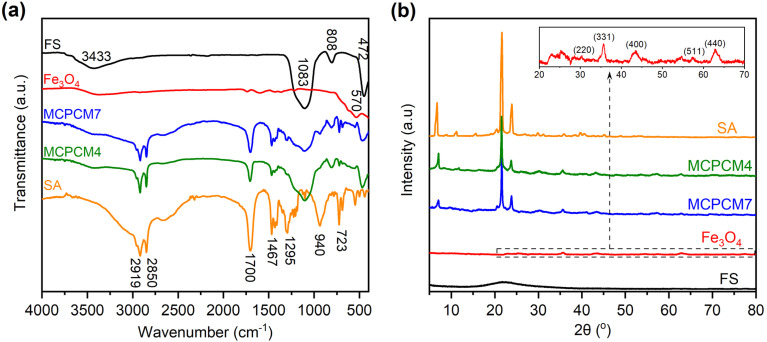
FTIR spectra (a) and XRD patterns (b) of FS, Fe_3_O_4_, SA, MCPCM4, and MCPCM7.

The FTIR spectra of two representative composites, MCPCM4 and MCPCM7, compared to those of pristine FS, Fe_3_O_4_, and SA, are shown in Fig. 3a. The characteristic absorption of FS and SA was thoroughly combined in the two MCPCMs, and no new peaks were detected. For example, those of FS could be observed at 3433 cm^−1^ due to vibrations of silanol groups (Si–O–H) and physically adsorbed water and at 1083, 808, and 472 cm^−1^ due to vibration modes of siloxane groups (Si–O–Si).^32^ Meanwhile, those of SA were found at 2919, 2850, and 1467 cm^−1^, corresponding to vibration modes of –C–H groups of the aliphatic backbone. In addition, the vibrations of –C=O groups were observed at 1700 cm^−1^, and those of –O–H groups were observed at 1295, 940, and within 3500–2500 cm^−1^, which were typical for the carboxylic acid group of SA.^33,34^ Note that the characteristic absorption of Fe_3_O_4_ was not clearly shown in the spectra of the two MCPCMs, although pristine Fe_3_O_4_ exhibited a peak at 570 cm^−1^ due to vibrations of Fe–O groups, attributed to the intrinsically low absorption of Fe–O groups.^35^ These results indicated that FS, SA, and Fe_3_O_4_ were physically composited without chemical reactions.

In addition to FTIR spectra, the XRD patterns of the corresponding materials are exhibited in Fig. 3b. The two composites showed the characteristic diffractions of pristine SA at 2*θ* values of 21.5 and 23.9°. Meanwhile, those of pristine FS and Fe_3_O_4_ NPs faintly appeared in the patterns of the two MCPCMs because of the amorphous nature of FS and the intrinsic weak intensities of Fe_3_O_4_ NPs. It is noted that pristine Fe_3_O_4_, as magnified in the inset of Fig. 3b, exhibited weak diffractions at 2*θ* values of 30.4, 35.7, 43.4, 57.3, and 62.8°, respectively, related to the crystal planes (220), (311), (400), (511), and (440) of magnetic Fe_3_O_4_ NPs (JCPDS card no. 019-0629),^36,37^ confirming successful preparation. Overall, the XRD results demonstrated an intact crystallization of SA in the composites, which was the basis for the heat storage/release.

### 3.2 Phase change properties

**Fig. 4:**
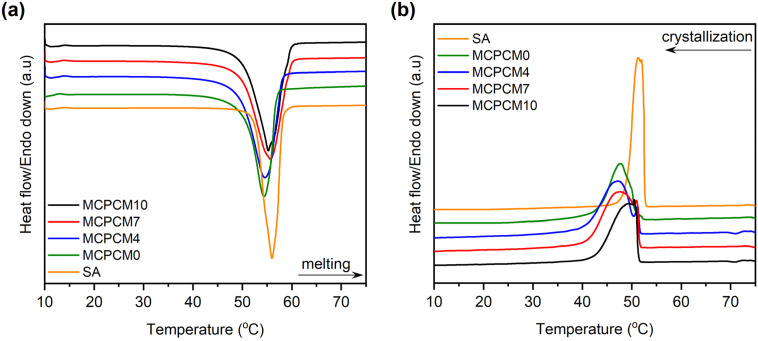
(a) Melting DSC curves and (b) crystallization DSC curves of SA and the prepared MCPCMs.

The phase change behaviors of the prepared composites compared to pristine SA were characterized using the DSC method. The obtained DSC thermograms are shown in Fig. 4 and the specific data are shown in Table 2. Compared to pristine SA, the composites exhibited slightly lowered melting temperatures (*T*_M_) of 2.2–2.5 °C and crystalline temperatures (*T*_C_) of 1.7–2.1 °C. Lower phase change temperatures were often observed when a PCM was confined in a porous support and were attributed to confinement effects in nanopores.^38,39^

**Table 2:** Phase change properties of SA and the prepared MCPCMs

	*T*_M_ (°C)	*T*_C_ (°C)	Δ*H*_M_ (J g^−1^)	Δ*H*_C_ (J g^−1^)	*F*_ME_ (%)	*F*_CE_ (%)	*η* (%)
SA	52.3	53.1	200.5	197.6			
MCPCM0	50.0	51.5	130.7	128.5	93.1	92.9	65.1
MCPCM4	49.8	51.4	131.8	129.0	93.9	93.3	65.5
MCPCM7	50.0	51.6	132.8	130.5	94.6	94.3	66.1
MCPCM10	50.1	51.0	133.9	132.1	95.4	95.5	66.8

Phase change enthalpy is essential in evaluating the thermal energy storage/release capacity for the MCPCMs. As shown in Table 2, pure SA exhibited a melting enthalpy (Δ*H*_M_) and a crystalline enthalpy (Δ*H*_C_) of 200.5 and 197.6 J g^−1^, respectively. Meanwhile, the prepared MCPCMs exhibited lower phase change enthalpies than pure SA. This phenomenon was facilely understood as, in the form of MCPCMs, SA was partially replaced by FS and Fe_3_O_4_, which had no contribution to the phase change enthalpies. In addition, it was reported that interactions, *e.g.*, hydrogen bonds (H-bonds), between PCMs and porous supports led to the formation of non-freezable layers of PCMs at the interfacial region. These layers could not crystallize and caused a lowered crystalline fraction of PCMs, especially for PCMs confined in tiny pores, thus decreasing phase change enthalpy.^40–42^ SA, a carboxylic acid, was known to form H-bonds with silanol groups on SiO_2_'s surfaces,^43,44^ and this could be another reason for the lower phase change enthalpies of MCPCMs. The crystalline fraction of a confined PCM is conventionally computed based on melting enthalpy, denoted as *F*_ME_ (%), using eqn (1).^38^ We additionally calculated the crystalline fraction based on crystallization enthalpy, denoted as *F*_CE_ (%), using eqn (2).1
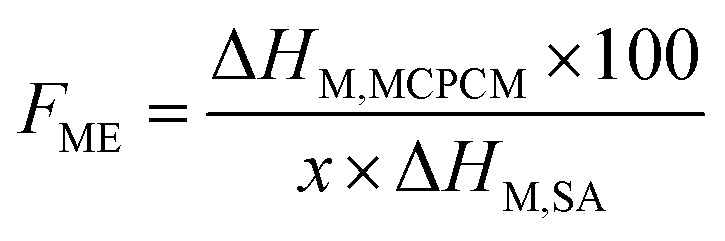
2
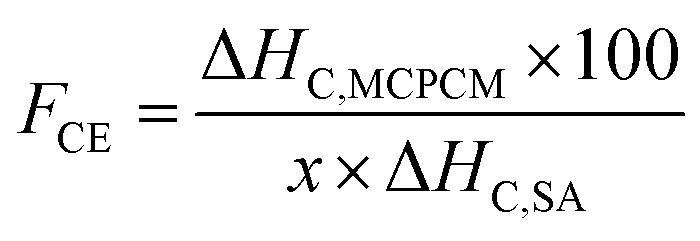
3
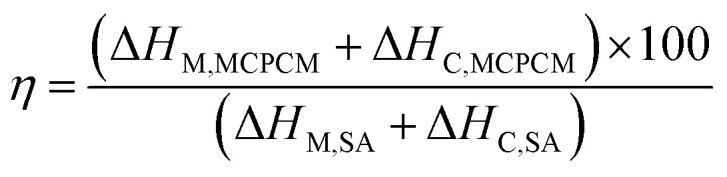
where Δ*H*_M,MCPCM_ and Δ*H*_M,SA_ are the melting enthalpies of the prepared MCPCM and pure SA, Δ*H*_C,MCPCM_ and Δ*H*_C,SA_ are the crystallization enthalpies of the prepared MCPCM and pure SA, respectively, and *x* is the mass ratio of SA in the composite. The crystalline fractions of SA in the MCPCMs were consistently below 100% and increased with increasing Fe_3_O_4_ contents, as calculated using both eqn (1) (93.1–95.4%) and eqn (2) (92.9–95.5%), as shown in Table 2, further confirming an incomplete crystallization. With more careful observation, it was seen that Δ*H*_M_ and Δ*H*_C_ of MCPCMs slightly increased with increasing Fe_3_O_4_ contents, ranging from 130.7 and 128.5 J g^−1^ for MCPCM0 to 133.9 and 132.1 J g^−1^ for MCPCM10, respectively, although they had the same SA content of 70%. This was attributed to the gradual decrease of FS content from 30 to 20% with the increasing Fe_3_O_4_ content from 0–10% in the MCPCMs, leading to a smaller number of interfacial H-bonds, thus promoting the crystalline fractions and phase change enthalpies. These effects could be consistently observed in the loading efficiencies (*η* (%)) of the MCPCMs (as calculated using eqn (3) (ref. 45)) that showed that *η* values increased from 65.1 to 66.8% with increasing Fe_3_O_4_ content from 0–10% (Table 2).

The *F* values of SA in the MCPCMs (93.1–95.4%) were relatively high and slightly affected the phase change enthalpies. Previous reports showed that SA confined in porous SiO_2_ materials with high surface areas including SBA-15 (931 m^2^ g^−1^)^46^ and tannic acid-templated SiO_2_ (471 m^2^ g^−1^)^47^ or SiO_2_ with tiny pores (2.74 nm)^48^ exhibited *F* values of only 2–64% due to a large number of interfacial H-bonds or limited free movement, which significantly decreased phase change enthalpies (Table 3). Therefore, a significant advantage of using FS with a moderate surface area (205 m^2^ g^−1^) and appropriate pore system (micro-, meso-, and macropores) compared to the other SiO_2_ materials was maintaining a high crystallinity for SA. As shown in Table 3, the *F* values of SA in this work are comparable to or even exceed those of SA confined in other non-SiO_2_ materials such as Mxene-graphene oxide (70.2%),^49^ UiO-66-1 (74.8%),^50^ boron nitride (84.3%),^51^ corn straw powder (93.2%),^52^ and ultrathin graphite sheets (94.1%),^53^ with the relevant *F* values shown in parentheses.

**Table 3:** Thermal properties of the prepared MCPCMs compared to those in previous reports

Composite	SA content (%)	Δ*H*_M_ (J g^−1^)	*F* (%)	Ref.
SA/SBA-15	50	36.3	35.5	46
SA/tannic acid-templated SiO_2_	70	108.8	65.0	47
SA/mesoporous SiO_2_	70	77.5	64.1	48
SA/Mxene-graphene oxide	96.1	139.3	70.2	49
SA/UiO-66-1	40	56.4	74.8	50
SA/boron nitride	88	149.3	84.3	51
SA/corn straw powder	50.0	98.47	93.2	52
SA/ultrathin graphite sheet	63.12	113.7	94.1	53
MCPCM4	70	131.8	93.9	This work
MCPCM7	70	132.8	94.6	This work
MCPCM10	70	133.9	95.4	This work

### 3.3 Thermal stability and conductivity

**Fig. 5:**
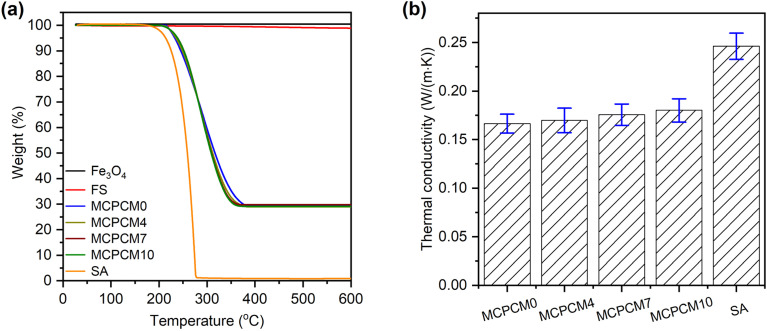
TGA curves of FS, Fe_3_O_4_, SA, and the prepared MCPCMs (a), and thermal conductivities of SA and the prepared MCPCMs (b).

The TGA method was used to characterize thermal stability, and the obtained TGA curves of the prepared MCPCMs, FS, SA, and Fe_3_O_4_ are exhibited in Fig. 5a. Compared to FS and Fe_3_O_4_ with negligible weight loss within the tested temperatures (up to 600 °C), SA, with an organic nature, showed almost complete weight loss within the temperature range of 225–278 °C. With the excellent thermal stability of FS and Fe_3_O_4_, the weight loss of MCPCMs could be attributed to the exclusive decomposition of SA. The prepared MCPCMs exhibited improved thermal stability compared to pure SA, characterized by the decomposition temperature range ascending to within 250–346 °C. The enhanced thermal stability was due to forces such as capillary forces, surface tension, and interfacial H-bonds that somewhat impeded the spillover of SA out of the FS porous networks before decomposition and was also observed in previous reports.^49,51,54^ In practical applications, the MCPCMs would undergo heat storage/release processes above and below their phase change temperatures (∼56 °C). Therefore, a high decomposition temperature of up to 250 °C assures high thermal stability for the MCPCMs during usage. In addition, the weight loss of MCPCMs was found to be 69.0–69.7%, consistent with the added amount of SA (70%) during the preparation.

Fig. 5b shows the thermal conductivity (TC) of the prepared MCPCMs and pure SA. Compared to pure SA with a low TC of 0.264 W (m^−1^ K^−1^), the prepared MCPCMs showed even lower TCs of 0.166–0.180 W (m^−1^ K^−1^). Of the constituents of MCPCMs, FS was known to have an ultralow TC of ∼0.05 W (m^−1^ K^−1^).^15^ Fe_3_O_4_ NPs also exhibited a low TC of 0.144–0.18 W (m^−1^ K^−1^), as reported elsewhere.^27,28^ Therefore, combining SA with FS and Fe_3_O_4_ NPs formed composites with decreased TCs. The TCs of the prepared MCPCMs slightly increased from 0.166 to 0.180 W (m^−1^ K^−1^) with an increase in the Fe_3_O_4_ contents from 0–10%. The rise in Fe_3_O_4_ content in the MCPCMs induced a corresponding decrease in FS content, and the TC accordingly increased because of the higher TC of Fe_3_O_4_ compared to FS. Overall, the low TC of MCPCMs has both advantages and disadvantages. On the one hand, it causes poor charging efficacy due to low heat transfer. On the other hand, the low heat transfer can prolong the heat release duration from the MCPCMs to targeted objects, which is required for applications such as thermotherapies.^9–11^ Thus, improving charging efficacy is necessary and worth studying to enhance the performance of MCPCMs in practical applications.

### 3.4 Leakage resistance and cycling durability

**Fig. 6:**
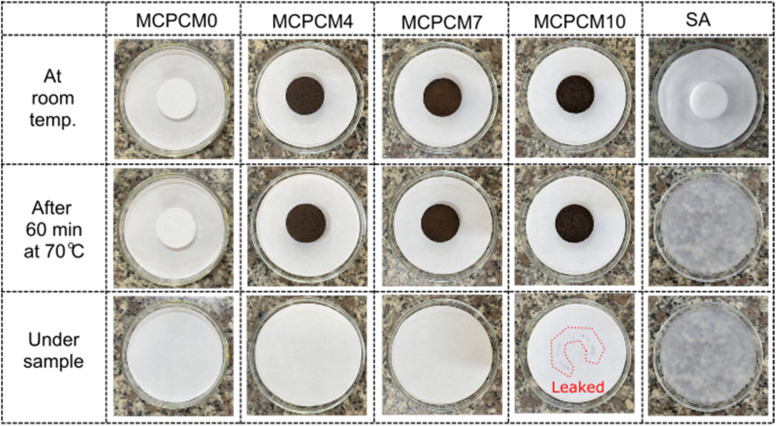
Leakage resistance of the prepared MCPCMs compared to pure SA.

The leakage resistance of the prepared MCPCMs compared to pure SA was evaluated by placing them on filter paper and treating them at 70 °C (∼17 °C above their melting points) in an oven for 60 min. Subsequently, the filter paper's areas underneath the samples were carefully observed for smears of leaked liquid SA, and the obtained digital photos are shown in Fig. 6. Compared to pure SA with complete liquidation and deformation, all the MCPCMs maintained their original shapes. However, they could only resist the leakage for samples with 0, 4, and 7% Fe_3_O_4_, while a slight leakage occurred for the sample with 10% Fe_3_O_4_. In the prepared MCPCMs, the SA content was fixed at 70%, and FS was the main component that provided porous networks for the confinement of SA. The increase in Fe_3_O_4_ fractions led to a corresponding decrease in FS content and, accordingly, a lowering of the SA loading capacity. With the rise in Fe_3_O_4_ content to 10%, the remaining FS content (20%) was insufficient to stabilize the liquid SA, resulting in leakage. These results indicated that the maximum Fe_3_O_4_ content should be limited to somewhere between 7 and 10% to maintain the stabilization of the MCPCMs. The sample MCPCM10 was therefore excluded from further studies.

**Fig. 7:**
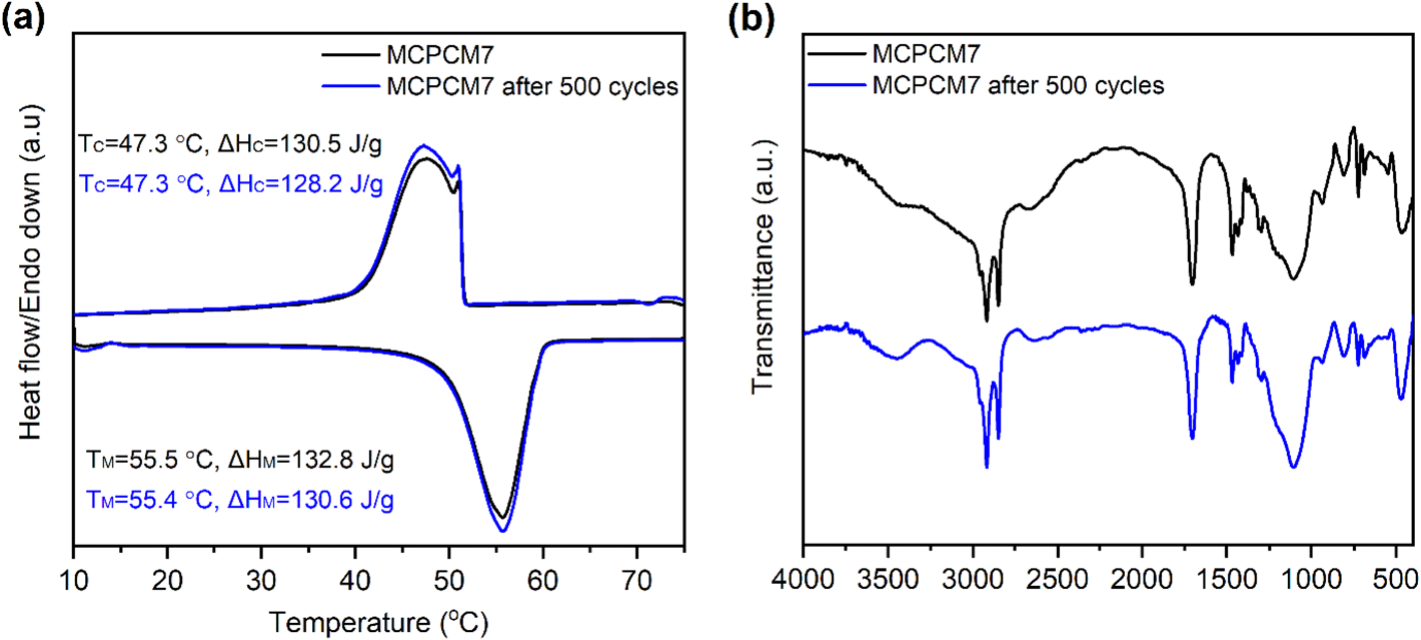
DSC curves (a) and FTIR spectra (b) of MCPCM7 at the first and 500th melting/crystallization cycles.

The sample MCPCM7 showed good leakage resistance, high Fe_3_O_4_ content, and high phase change enthalpies. Thus, it was considered an optimal composite in this work and evaluated for cycling durability with 500 melting/crystallization cycles. The cycling durability characterized by DSC and FTIR of MCPCM7 is shown in Fig. 7. The composite showed negligible differences in DSC curves before and after the multiple thermal cycles, almost leading to a monotony in phase change temperatures (Fig. 7a). The Δ*H*_M_ and Δ*H*_C_ of the 500th cycled sample were 130.6 and 128.2 J g^−1^, only 1.6 and 1.8% lower than those of the 1st cycle, respectively. The FTIR spectra (Fig. 7b) also exhibited negligible differences in positions and intensities of the characteristic absorption peaks of the samples before and after the cycling experiment. The DSC and FTIR results proved excellent durability for the MCPCM after repeated melting/crystallization cycles, making it applicable for long-term utilization.

### 3.5 Magnetothermal conversion and storage

**Fig. 8:**
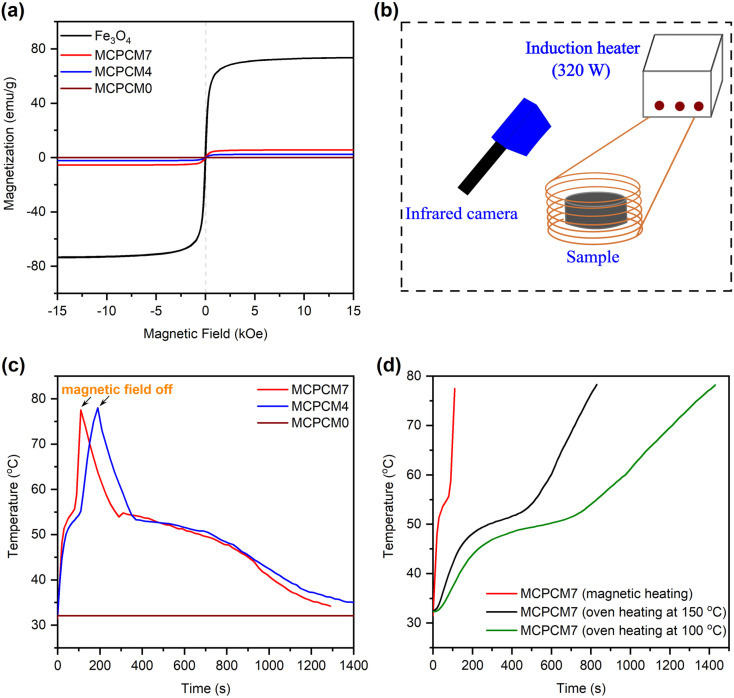
VSM curves of MCPCM0, MCPCM4, and MCPCM7 compared to pristine Fe_3_O_4_ (a), illustration of magnetothermal conversion apparatus (b), temperature–time curves of MCPCM0, MCPCM4, and MCPCM7 during the magnetothermal conversion test (c), and temperature–time curves of MCPCM7 heated by magnetic power and ovens at different temperatures (d).

The VSM curves of pristine Fe_3_O_4_ and the prepared MCPCM0, MCPCM4, and MCPCM7 are shown in Fig. 8a. The prepared MCPCM4 and MCPCM7 exhibited saturation magnetizations of 2.3 and 5.6 emu g^−1^ respectively, which were inherited from the presence of Fe_3_O_4_ with a high saturation magnetization of 73.7 emu g^−1^. In addition, similar to pristine Fe_3_O_4_, they showed very small coercivities and remanences. These results demonstrated that the superparamagnetism of Fe_3_O_4_ was maintained in the prepared MCPCM4 and MCPCM7. Meanwhile, MCPCM0 showed no saturation magnetization, which was readily understood by its non-magnetic nature.

The magnetothermal conversion performance of the prepared MCPCM0, MCPCM4, and MCPCM7 was evaluated by placing them in an inductor with a magnetic intensity of 0.056 mT and an alternating magnetic field frequency of 153 kHz. The temperature change during the experiment was recorded using an infrared camera, as illustrated in Fig. 8b, and the obtained temperature evolution curves are shown in Fig. 8c. Under an applied magnetic field, the temperatures of MCPCM4 and MCPCM7 rapidly increased from 32 °C to 78 °C in 170 and 110 s, respectively. The temperature evolution was directly proportional to the Fe_3_O_4_ content in the materials. Meanwhile, the temperature of MCPCM0 was kept intact at 32 °C during the experiment due to its non-magnetism. The results demonstrated a good magnetothermal conversion of MCPCM4 and MCPCM7, which was facilitated by the effects of Néel relaxation (involving the magnetic dipole rotation within the particle) and/or Brownian relaxation (involving the entire particle's rotation in response to a magnetic field) of magnetic materials.^26,55^

The magnetothermal conversion ability provides MCPCM4 and MCPCM7 with effective heating for thermal storage. With a more thorough analysis, the temperature evolutions during the magnetothermal conversion experiment of MCPCM4 and MCPCM7 (Fig. 8c) can be divided into three steps. In the first step (32–51 °C), the magnetic power was converted into thermal energy and stored in the MCPCMs as sensible heat, followed by fast temperature evolutions. This step occurred below the melting point of confined SA, which meant that the confined SA was in a solid state. The Fe_3_O_4_ NPs were accordingly immobilized by the surrounding solids, and Brownian relaxation, which was related to the rotation of entire Fe_3_O_4_ NPs, could not occur. Thus, Néel relaxation was the dominant heating mechanism in this step. The second step (51–55 °C) was established with the conversion and storage of magnetic power in the form of latent heat during the melting of SA in the MCPCMs, forming temperature platforms. In the last step (55–78 °C) after the completion of SA melting, the magnetic power was again stored as sensible heat, leading to fast temperature evolutions. The second and final steps were accompanied by the liquidization of confined SA. Thus, both Néel and Brownian relaxation possibly contributed to the heating mechanism. After the magnetic power was turned off, the temperatures of MCPCM4 and MCPCM7 first went down and then established platforms within ∼53–45 °C due to heat release during the crystallization of SA.

The magnetic heating performance of MCPCM7 was further compared to traditional oven heating at 100 and 150 °C, and the obtained temperature evolutions are shown in Fig. 8d. Compared to magnetic heating (110 s), oven heating at 100 °C (1430 s) and 150 °C (830 s) showed heating rates 13.0 and 7.5 times slower to increase the temperature from 32 to 78 °C, respectively (with the durations shown in parentheses). The fast heating performance benefited practical applications. Overall, it can be concluded that MCPCM4 and MCPCM7 possessed effective magnetothermal conversion and storage/release properties.

### 3.6 Practical thermotherapy performance

**Fig. 9:**
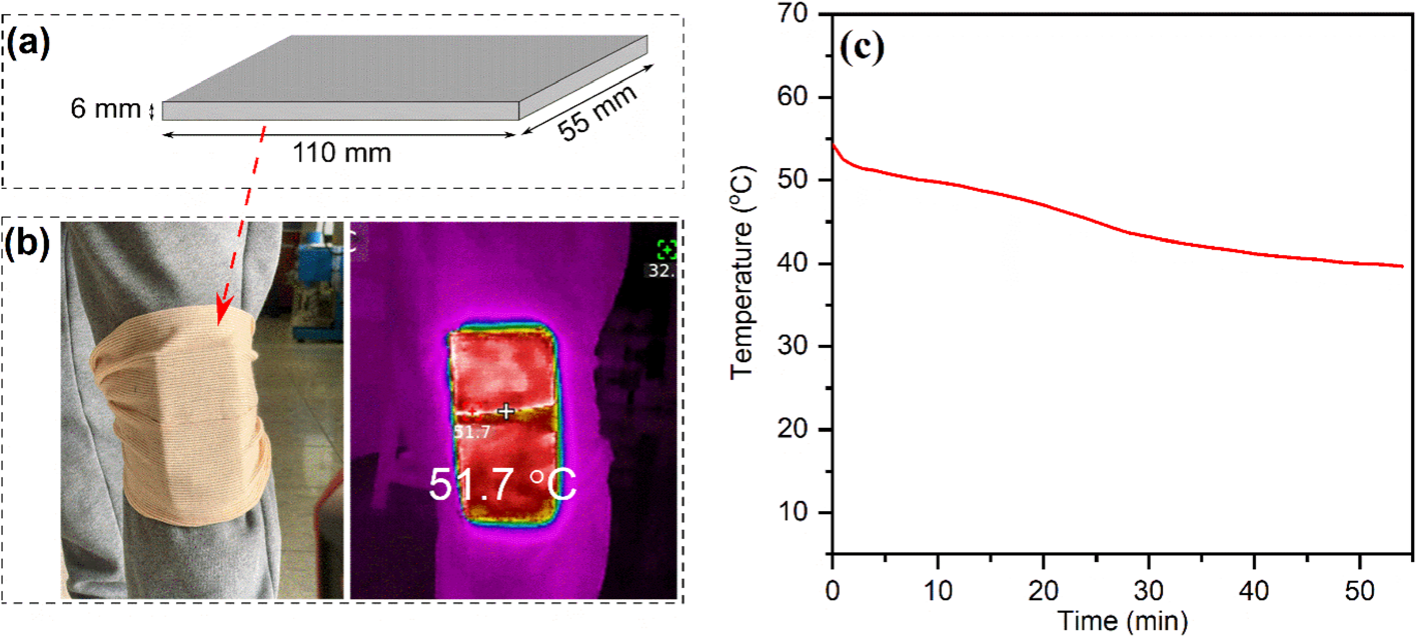
Illustration of the heat pad prepared from MCPCM7 (a), digital and infrared images of the heat pad attached to the knee of a volunteer (b), and a temperature–time curve recorded at the interface between the heat pad and the knee (c).

The practical thermotherapy performance was examined for MCPCM7. The composite (40 g) was compressed into a heat pad (110 × 55 × 6 mm, Fig. 9a) and heated to ∼65 °C (∼10 °C above its melting temperature) for heat storage. Note that the heating was conducted using an oven because the heat pad was too large to place in the small-scale inductor used in the magnetothermal conversion experiment (Section 3.5). However, the heating methods (magnetism or oven) for heat storage would not affect the thermotherapy performance which was measured during the heat release. After heating, the heat pad was left to cool naturally to ∼55 °C and then attached to the knee of a volunteer for the illustration of rheumatic arthritis thermal treatment, as shown in Fig. 9b. The temperature variation at the interface between the pad and the knee was monitored using a thermocouple, and the obtained temperature–time curve is exhibited in Fig. 9c. When the temperature of the heat pad cooled to ∼52 °C, the temperature-decreasing rate was slowed down, forming a temperature platform until ∼42 °C due to the crystallization of SA. A total duration of 35 minutes was recorded for heat release between 52 and 42 °C, in which the first 7 minutes were in the temperature range of 52–50 °C, and the last 28 minutes were in the temperature range of 50–42 °C. This performance meets the requirements for both high-level thermotherapy (50–55 °C, 4–6 minutes) and middle-level thermotherapy (40–50 °C, 15–60 minutes).^3,4^ Thus, the heat pad can be used as a bi-functional thermotherapy method. Overall, the good performance made MCPCM7 highly promising in practical thermotherapy applications. It is noted that a large-scale inductor should be fabricated to be able to charge the heat pad magnetically for a fast heating rate.

## 4. Conclusions

In summary, novel magnetic composite phase change materials for thermotherapy were prepared using SA as a heat storage/release medium, FS as a matrix, and Fe_3_O_4_ NPs as magnetothermal conversion additives. These materials were physically composited and the crystallization properties of SA were maintained in the MCPCMs. The prepared MCPCMs exhibited high energy storage capacities (∼132 J g^−1^) and crystallization temperatures of ∼51 °C, which were suitable for thermotherapy. They showed low TCs of 0.166–0.180 W (m^−1^ K^−1^), which were even lower than the value of 0.264 W (m^−1^ K^−1^) of pure SA due to the low TCs of FS and Fe_3_O_4_ NPs. MCPCM7 consisting of only 23% FS, 70% SA, and 7% Fe_3_O_4_ NPs exhibited excellent leakage resistance of liquid SA owing to the high porosity of FS. It possessed superparamagnetism with a saturation magnetization of 5.6 emu g^−1^, which led to an accelerated magnetothermal conversion. Indeed, it exhibited magnetic heating rates 13.0 and 7.5 times faster than heating by using ovens at 100 and 150 °C, respectively. A practical test confirmed that a heat pad prepared using MCPCM7 met the criteria for high and middle-level thermotherapy. These results provided a new strategy to prepare long-duration thermotherapy materials with an effective heating rate performance.

## Data availability

The data supporting this article have been included as part of the ESI.

## Conflicts of interest

There are no conflicts to declare.

## Supplementary Material

NA-OLF-D5NA00133A-s001
