# Supplementary material for: Stearic acid/fumed silica/Fe3O4 composite phase change materials with low thermal conductivities and magnetically accelerated heating performance for wearable thermotherapy
Source: Nanoscale Adv. 2025 Apr 9;7(11):3236–46. doi: 10.1039/d5na00133a (PMC11979488; doi:10.1039/d5na00133a)
Supplement: NA-007-D5NA00133A-s001 [file NA-007-D5NA00133A-s001.pdf]

Supplementary Information (SI) for Nanoscale Advances.
This journal is © The Royal Society of Chemistry 2025

Supplemental Information

Stearic Acid/Fumed Silica/Fe_3O_4 Composite Phase Change Materials with Low Thermal Conductivities and Magnetically Accelerated Heating Performance for Wearable Thermotherapy

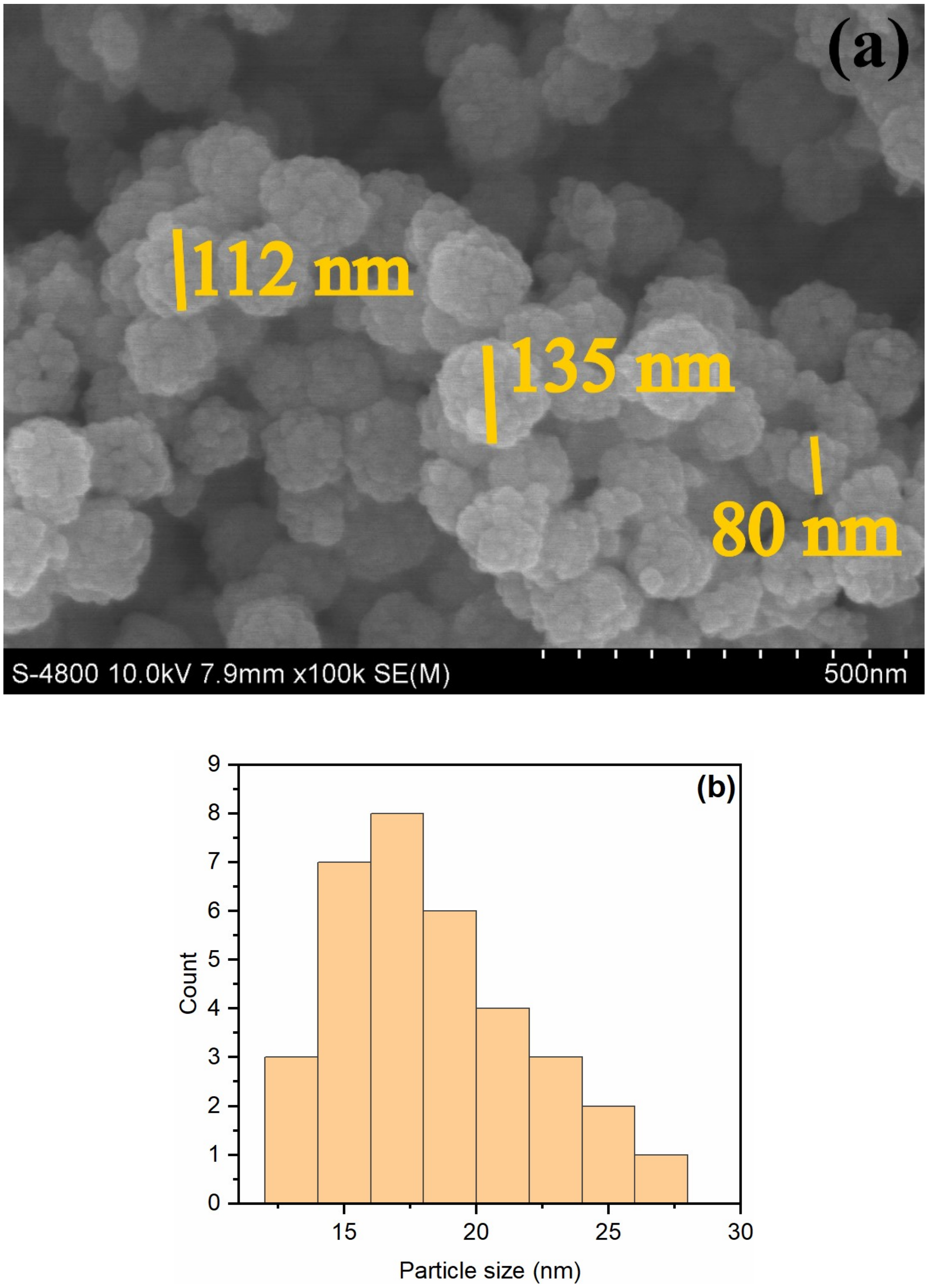

Figure S1. (a) SEM image and (b) particle size distribution of the prepared Fe_3O_4 NPs.
